# Adapting an equity-focused implementation process framework with a focus on ethnic health inequities in the Aotearoa New Zealand context

**DOI:** 10.1186/s12939-023-02087-y

**Published:** 2024-01-27

**Authors:** Papillon Gustafson, Michelle Lambert, Karen Bartholomew, Mihi Ratima, Yasmin Abdul Aziz, Lisa Kremer, Adam Fusheini, Peter Carswell, Rachel Brown, Patricia Priest, Sue Crengle

**Affiliations:** 1 Ngāi Tahu Māori Health Research Unit, Division of Health Sciences, University of Otago, Dunedin Campus, PO Box 56, Dunedin, Aotearoa New Zealand 9054; 2 Te Whatu Ora Waitematā and Te Toka Tumai Auckland, Auckland, Aotearoa New Zealand; 3 Taumata Associates, Hāwera, Aotearoa New Zealand; 4 Preventive and Social Medicine, University of Otago, Dunedin Campus, Dunedin, Aotearoa New Zealand; 5 Synergia Ltd, Auckland, Aotearoa New Zealand; 6 National Hauora Coalition, Auckland, Aotearoa New Zealand

**Keywords:** Health equity, Implementation, Framework, Ethnic inequities, Māori, Indigenous, Aotearoa New Zealand, Te Tiriti o Waitangi, Process model

## Abstract

**Background:**

Health intervention implementation in Aotearoa New Zealand (NZ), as in many countries globally, usually varies by ethnicity. Māori (the Indigenous peoples of Aotearoa) and Pacific peoples are less likely to receive interventions than other ethnic groups, despite experiencing persistent health inequities. This study aimed to develop an equity-focused implementation framework, appropriate for the Aotearoa NZ context, to support the planning and delivery of equitable implementation pathways for health interventions, with the intention of achieving equitable outcomes for Māori, as well as people originating from the Pacific Islands.

**Methods:**

A scoping review of the literature to identify existing equity-focused implementation theories, models and frameworks was undertaken. One of these, the Equity-based framework for Implementation Research (EquIR), was selected for adaptation. The adaptation process was undertaken in collaboration with the project’s Māori and consumer advisory groups and informed by the expertise of local health equity researchers and stakeholders, as well as the international implementation science literature.

**Results:**

The adapted framework’s foundation is the principles of Te Tiriti o Waitangi (the written agreement between Māori rangatira (chiefs) and the British Crown), and its focus is whānau (extended family)-centred implementation that meets the health and wellbeing aspirations, priorities and needs of whānau. The implementation pathway comprises four main steps: implementation planning, pathway design, monitoring, and outcomes and evaluation, all with an equity focus. The pathway is underpinned by the core constructs of equitable implementation in Aotearoa NZ: collaborative design, anti-racism, Māori and priority population expertise, cultural safety and values-based. Additionally, the contextual factors impacting implementation, i.e. the social, economic, commercial and political determinants of health, are included.

**Conclusions:**

The framework presented in this study is the first equity-focused process-type implementation framework to be adapted for the Aotearoa NZ context. This framework is intended to support and facilitate equity-focused implementation research and health intervention implementation by mainstream health services.

**Supplementary Information:**

The online version contains supplementary material available at 10.1186/s12939-023-02087-y.

## Background

Achieving equitable health outcomes requires approaches that recognise and address the differing levels of advantage people have in society [1, 2]. Health inequities are the avoidable, unfair and unjust differences in health between groups of people, which may be defined based on demographic, geographic or socioeconomic factors [1, 2]. Ethnic health inequities are those experienced by population groups defined by shared geographic origin and ancestry, and is inclusive of groupings based on “race” that are still commonplace in some jurisdictions [3]. Ethnic health inequities are well-documented globally and many minoritised ethnic groups have poorer health compared to the majority [4–9].

In Aotearoa New Zealand (NZ), Māori (the Indigenous peoples) and Pacific peoples experience marked health inequities compared to the majority European population. Life expectancy for Māori is on average seven years lower than for non-Māori [10]. Similarly, life expectancy for Pacific peoples is on average six years lower than for non-Māori, non-Pacific peoples [11]. Māori and Pacific peoples are more than twice as likely to die from potentially avoidable causes as non-Māori, non-Pacific people [12]. There are multiple interacting causes of health inequities, including differential access to and through affordable, quality, culturally safe healthcare, and differential exposure to the determinants of health, e.g. education, employment, income, housing [13, 14]. While racism is a determinant of health, it also characterises colonial societies and is a central underlying cause of health inequities for Indigenous peoples [15–17]. For Māori, like many other Indigenous populations globally, systematic health inequities are inextricably linked to the historical and contemporary impacts of colonisation [14, 18, 19]. Māori Health providers in Aotearoa NZ provide ‘by Māori for Māori’ health services to Māori and others using a kaupapa Māori model with the aim to reduce these impacts.

Uptake of health interventions by Māori and other minority groups in Aotearoa NZ is affected by a variety of factors, including social determinants like ethnicity, education and income, [20] as well as patient-clinician interactions and cultural competencies of clinicians and the system [21]. Māori and Pacific peoples are less likely to receive interventions than other ethnic groups, with evidence of this in cancer screening [22], diabetes screening during pregnancy and postpartum [23, 24], cardiovascular disease risk assessment [25], vaccinations [26, 27] and evidence-based treatments [28–30]. We have defined ‘intervention’ as a broad range of innovations intended to improve human health, including treatments, procedures, programmes and services. Ensuring that implementation pathways are designed to enable equity in outcomes and/or reduce inequity is key to mitigating the inequities associated with intervention implementation.

Implementation science studies the influencing factors, methods and processes for systematically promoting intervention uptake into routine practice [31]. Theories, models and frameworks (TMFs) are used in implementation research and practice to explore and identify the factors that influence implementation. They provide guidance for implementation processes, evaluation of implementation outcomes, definition of the scope of implementation research and which constructs to measure. TMFs also support the interpretation of implementation research findings and development of the empirical evidence base [32, 33]. Increasingly, TMFs are being developed or adapted to have an equity focus, including identifying the factors that influence equitable implementation, establishing equity-focused implementation processes, and evaluating implementation outcomes with an equity lens and looking at the process of addressing unequitable outcomes [34]. There have been many years of health research that has excluded indigenous communities, and by doing so, this research has contributed to health inequities. By applying an equity lens, we are able to use this approach to ensure that we do not create a model that perpetuates health inequities [35].

Improving health equity has been identified as a priority by the Aotearoa NZ government [36]. In 2014, the National Science Challenges (NSCs) were established to fund mission-based research addressing eleven science-based issues using collaborative approaches, stakeholder engagement and participation, and with the involvement of Māori and mātauranga (Māori knowledge) [37]. One of these Challenges, Healthier Lives, has the mission of reducing the burden of non-communicable diseases and increasing equitable health outcomes in NZ [38]. In this article, we present one component of a project in the Healthier Lives NSC that aims to support the health system to implement interventions and improve health equity.

Adopting a systematic approach to the implementation of interventions is relatively uncommon in Aotearoa NZ, however, utilisation of implementation science approaches to address health inequities is increasing [39–41]. In 2017, as part of another Healthier Lives NSC project, Oetzel, Scott and colleagues published the He Pikinga Waiora Implementation Framework to support effective and culturally-appropriate chronic disease intervention implementation for Māori and other indigenous communities, with a focus on co-design [40]. The Framework’s authors proposed that He Pikinga Waiora could be used as a planning tool to support intervention development and implementation for researchers, funders and health organisations [40]. He Pikinga Waiora has been operationalised in several studies, including co-designing and evaluating interventions in Māori communities [42–46].

As implementation science approaches become more common in Aotearoa NZ, an identifiable gap in the literature is step-by-step guidance for researchers and practitioners through equity-focused implementation pathway design and delivery. In implementation science, process models provide guidance for how to translate research into practice, usually as a series of steps or stages [19, 29]. The aim of this study was to develop an equity-focused implementation framework, appropriate for the Aotearoa NZ context, to support the planning and delivery of equitable implementation pathways for health interventions, with the intention of achieving equitable outcomes. The focus of the development of this framework is on ethnic health inequities, particularly for Māori, as well as its potential application for addressing health inequities for Pacific peoples and other minoritised ethnic or population groups. This framework is primarily intended to support and facilitate equity-focused implementation research and health intervention implementation by ‘mainstream’ health services/providers (as opposed to Indigenous health providers).

## Methods

The framework development process involved six key steps.

### Step 1: literature review on equity-focused implementation science TMFs

A scoping review of the literature on equity-focused implementation science TMFs was conducted, including TMF components, extent of equity and systems focus and operationalisation. A protocol for the review and the results have been published previously [34, 47].

### Step 2: interviews with stakeholders and researchers in Aotearoa NZ

As a part of a broader study, twelve lead or principal investigators who had successfully developed or trialled health interventions and implementation research and thirteen health service leaders, including those who worked in service and management roles in District Health Boards, Māori Health leads, General Practitioners and managers/directors of non-government organisations, were interviewed about factors they considered to be key in the implementation of interventions that improved equity. The interviews were 40–65 min long and focused on questions related to how Māori are considered in the design and delivery of interventions, and how equity is explicitly considered. The data were analysed using a thematic approach, with initial deductive coding based on a framework developed from the Health Equity Implementation Framework [48] domains and early insights gathered from the interviews. The second inductive stage of coding was guided by Gioia et al. [49] in which team members (led by PC) engaged in the theory building process, identifying themes that helped explain the implementation process for interventions aiming to support equity. Through this process, the first order concepts were examined for commonalities; where commonalities were found they were grouped. This process continued until theoretical saturation was reached [50]. The analysis of the interviews led to 39 first order concepts. Applying Gioia et al.’s method reduced this to fourteen different second order themes, grouped within one of four third order domains. These are presented in Additional file 1.

### Step 3: selection of a TMF to adapt

Steps 3 – 5 took place at a single-day meeting with members of the research team (SC, KB, PP, AF, ML, PG, YAA), which included those with expertise in health equity (SC and KB), Māori health (SC, ML) and the Aotearoa NZ health system (SC, KB, PP). The research team reviewed the TMFs identified through the literature search to determine whether adaptation of an existing TMF or development of a TMF de novo was most appropriate. All team members present evaluated the TMF options available and assessed their appropriateness for this study requirements before returning to discuss and decide the appropriate way to proceed, Based on this consensus process the Equity-based Framework for Implementation Research (EquIR), developed by Eslava-Schmalbach et al. [51], was selected for adaptation.

The justification for selecting the EquIR for adaptation was multi-factorial. Firstly, the EquIR is an equity-focused, process-type implementation framework that provides guidance through the entire implementation pathway from design through to evaluation of implementation outcomes. Secondly, the EquIR centres people as the focus of equity-focused implementation research. Thirdly, the framework incorporates the broader contextual factors that are known to influence implementation and health equity directly and indirectly, across a diverse range of sectors and determinants (social, economic, political and commercial). Finally, it provides a visual representation of the iterative process of design, implementation and evaluation in relation to the target population’s (hereafter referred to as the 'priority population’) health status and the intended outcomes of the intervention being implemented.

### Step 4: adaptation of the framework

The adaptation process began by reviewing each step and sub-step of the agreed model sequentially using the expertise of the research team to evaluate its appropriateness and any modifications that were required to meet the study purpose and reflect the needs of the Aotearoa NZ health context. In addition to the He Pikinga Waiora Implementation Framework [40], other implementation TMFs consulted through this process included two determinant frameworks (the Health Equity Implementation Framework (HEIF) [48] and the Consolidated Framework for Implementation Research (CFIR) [52]) and two evaluation frameworks (the Reach, Effectiveness, Adoption, Implementation, and Maintenance (RE-AIM) framework [53] and the equity-focused adaptation of Proctor et al.’s implementation framework [54]).

There were several aspects of the framework that we sought to adapt to address the aim of this study. First, the research-focused language was identified as a potential limiting factor for use of the framework in non-research settings. A key aim of our research was to establish a cyclical framework that could be utilised across academic and service settings, and language was identified as a key aspect that could influence its usability. Second, there was a lack of overt consideration of, or prompt for, community engagement and leadership, particularly in the implementation planning phase. Third, absence of ‘Implementation’ as a clear step; the ‘Implementing EquIR’ step focuses on designing and defining elements of the research programme [51]. However, having an ‘action’ step where implementation occurs was identified as useful to encourage active, equity-focused monitoring and feedback while the intervention is being implemented. Fourth, there was no assessment of intervention effectiveness. Incorporating an evaluation of intervention effectiveness in this framework was identified as a key approach to encourage those planning and designing interventions to incorporate assessments of implementation effectiveness alongside intervention effectiveness. Other implementation frameworks, for example RE-AIM, incorporate effectiveness as an outcome measure. Fifth, the inclusion of ‘universal health coverage.’ This term is not relevant within Aotearoa NZ as there is a publicly funded health system that provides the majority of healthcare; although it is acknowledged that cost can be a barrier to accessing primary healthcare services due to the existence of a co-payment scheme [55].

### Step 5: mapping emergent themes from researcher and stakeholder interviews against the adapted framework

The emergent themes and sub-themes from analysis of interviews with researchers and stakeholders in the Aotearoa NZ health system context were mapped against the first iteration of the framework to ensure that these were represented.

### Step 6: consultation and iterative revision

The first iteration of the framework was presented to the project Kāhui (Māori Advisory Group) and Consumer Advisory Group. The Kāhui governance group is all Māori and comprised of experts in Māori health research and service provision, as well as Iwi (tribal group) representation. The Consumer Advisory Group comprised of majority Māori health service consumers. These groups are tasked with the responsibility of being co-design partners. They are offered the opportunity to contribute to the design of the tools being developed during regular joint advisory group meetings with the research team. The members of these groups were selected for their experience as practitioners, researchers or consumers in Aotearoa NZ health services. They are governed by Terms of Reference agreed to by each member before their inclusion in the respective groups. These groups provided feedback on the framework components and design, which was then incorporated into a revised version. The second iteration of the framework was presented at a research team workshop where further refinements were made. The third iteration of the framework was developed based on these refinements and additional feedback provided by a research team member with expertise in Māori health research (MR). This version was presented to the Kāhui and Consumer Advisory Group for their input and feedback.

## Results

**Fig. 1:**
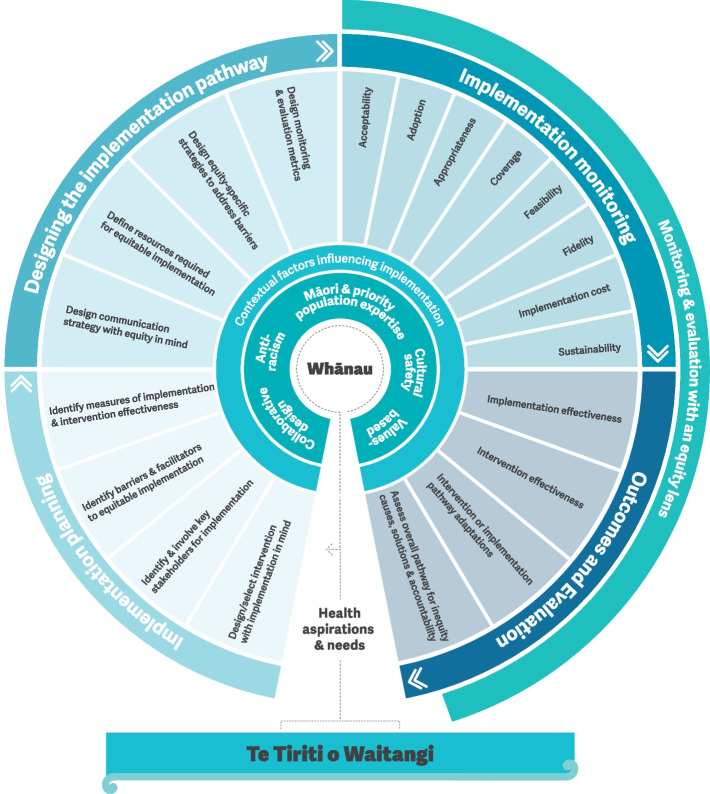
Equity-focused implementation framework to support the equitable implementation of health interventions, programmes and services in Aotearoa NZ

The framework (see Fig. 1) is comprised of five key elements, each is described in further detail below:

1. *The foundation* is the articles of Te Tiriti o Waitangi (The Treaty of Waitangi); all aspects of equitable implementation are informed by interpretations of the articles of Te Tiriti.
2. *The focus* is whānau (extended family)-centred implementation to achieve equitable outcomes for whānau as a group and for individuals within the context of whānau. The intent is that implementation meets the health and wellbeing aspirations, priorities and needs of whānau.
3. *The core constructs* are the five key elements that drive equitable implementation and inform each step along the implementation pathway: collaborative design, anti-racism, Māori and priority population expertise, cultural safety and values-based.
4. *The contextual factors *are the social, economic, commercial and political determinants of health that impact on intervention implementation and health equity.
5. *The implementation pathway *includes four main steps: Implementation Planning, Designing the Implementation Pathway, Implementation Monitoring, and Outcomes and Evaluation.

### The foundation

The foundation of this framework is Te Tiriti o Waitangi, which is the written agreement between Māori rangatira (chiefs) and the British Crown, signed in 1840 [56]. In accordance with the principle of contra preferendum the Māori language version of Te Tiriti takes precedence [57]. An interpretation of the articles of Te Tiriti is expressed in five principles articulated by the Courts and the Waitangi Tribunal [57, 58] that apply across the health and disability system and are, therefore, foundational to this implementation framework. The Tiriti principles, their meaning within the health and disability system, and examples of how they may be operationalised within the implementation pathway are outlined in Table 1. This is important because within the Aotearoa NZ health system, health and disability are under the same government appointed commissioner.

**Table 1:** Te Tiriti o Waitangi principles for the Aotearoa NZ health and disability system, their meaning and implications for implementation [59, 60]

Treaty principle	Meaning	Implications for implementation
Tino rangatiratanga	Provision for Māori self-determination and mana motuhake in the design, delivery and monitoring of health and disability services	Opportunities for Māori leadership and self-determination, including alignment to Māori-defined aspirations and priorities, are maximised at each step of the pathway
Equity	Commitment to achieving equitable health outcomes for Māori	Proactive focus on how Māori health equity has been fully considered and addressed in each step of the pathway
Active protection	Acting, to the fullest extent practicable, to achieve equitable health outcomes for Māori. This includes ensuring that the Crown, its agents and its Treaty partner under Te Tiriti are well informed on the extent, and nature, of both Māori health outcomes and efforts to achieve Māori health equity	Support Māori community readiness to enable the full and authentic engagement of whānau and communities in the implementation process
Options	Provision and proper resourcing for kaupapa Māori health and disability services. Furthermore, the Crown is obliged to ensure that all health and disability services are provided in a culturally appropriate way that recognises and supports the expression of hauora Māori models of care	Ensure inclusion of Māori expertise to more fully understand Māori health inequity and needs, and enable implementation that is informed by mātauranga Māori (including Māori models of healthcare), conducive to kaupapa Māori approaches, has a decolonisation orientation and is impactful. Ensure organisational readiness, such as cultural safety capacity
Partnership	Working in partnership with Māori in the governance, design, delivery and monitoring of health and disability services – Māori must be co-designers, with the Crown, of the primary health system for Māori	Work in genuine partnership with Māori throughout the implementation process. Practice co-design with Māori communities

The intention of this framework is to support mainstream services, as opposed to Indigenous (kaupapa Māori) providers, to equitably implement interventions, as an inherent outcome of kaupapa Māori provision (i.e. ‘by Māori, for Māori, with Māori’) is reduced inequities for Māori. Application of the framework in mainstream settings, therefore, relies on putting Te Tiriti principles into practice to drive equitable implementation for Māori.

### The focus

As conceptualised by Whānau Ora (a culturally grounded, holistic approach to improving wellbeing [61]), the whānau focus of the framework seeks to improve the wellbeing (and equity outcomes) of whānau as a group, and individuals within the context of whānau. Further, the implementation pathway for the intervention should be user-centred, i.e. informed by those with lived experience, and designed or adapted for the context where implementation will occur. Context in this instance refers to the characteristics and circumstances that are relevant to a particular implementation process, including the environmental setting, resource availability and the people involved (i.e. those involved in implementing the intervention and the recipients of the intervention) [52, 62].

### The core constructs

The core constructs were identified by the research team, and informed by interviews with stakeholders and researchers, local implementation science literature and feedback from the project Kāhui and Community Advisory Group. These key elements, described in Table 2, are important drivers of equitable implementation within an Aotearoa NZ context.

**Table 2:** Core constructs driving equitable implementation pathways in the Aotearoa New Zealand context

Core construct	Description
Collaborative design	The implementation pathway design is led by, or occurs in partnership with, the community of interest (e.g. population experiencing health inequities). Participatory processes are embedded, with the type of collaborative approach utilised (co-creation, co-design or co-production [63]) determined by the type of intervention and the implementation context. *Co-creation* is an overarching principle that describes a collaborative approach to problem identification and solving, solution implementation and evaluation between diverse stakeholders who are actively engaged and participating at all project stages [63]. *Co-design* describes the collaborative process between stakeholders to design solutions to pre-specified problems [63]. *Co-production* describes stakeholder engagement in the implementation of previously determined solutions to previously determined problems, with a focus on how best to use existing assets and resources [63]
Anti-racism	There is explicit recognition of and efforts to address ethnicity or “race”-based prejudice and discrimination at the level of individuals, institutions and structures [64–66]. This includes the examination of power and privilege by individuals and institutions and how this influences intervention design and implementation, and the re-distribution of power, privilege, resources and opportunities to address racism and achieve health equity [40, 64, 66, 67]
Māori and priority population expertise	Implementation pathway design and delivery is informed by Māori expertise and, if the intervention is being implemented in a priority population (e.g. Pacific peoples or other minoritised ethnic or population groups), the expertise of that group
Cultural safety	Self-examination by individuals and organisations involved in the provision of healthcare about the impact of their own culture and biases, assumptions, attitudes, stereotypes and prejudices on clinical interactions and healthcare provision, and actions to address these, with a clear goal of achieving health equity [68]
Values-based	Explicitly articulate and reflect the guiding principles and beliefs of the intervention’s priority or target population (population experiencing health inequity), as determined by that population

### The contextual factors

Contextual factors are the social, economic, commercial and political determinants of health that impact implementation processes and outcomes and health equity [48]. These influences must be considered in relation to where and to whom the intervention will be delivered and the potential influences on equitable implementation and implementation success.

### The implementation pathway

#### Step 1: Implementation planning

##### Design/Select intervention with implementation in mind

This framework assumes that an intervention has already been designed or selected for implementation. This includes new interventions designed to address health inequities, interventions that are being implemented in a new context, and population-wide interventions where there is potential for inequitable implementation. There are various tools available to assist with equity-focused intervention design in Aotearoa NZ, including the Health Equity Assessment Tool (HEAT) [69], the Equity of Health Care for Māori framework [70], Whānau Ora Health Impact Assessment [71] and the He Pikinga Waiora Implementation Framework [40].

Key considerations prior to implementation include:

- Reach: Who is invited, included, participating and engaged in the intervention design and implementation process, and who may be missing [54]. The people who are invited, included, participating and engaged should mirror the population intended to benefit from the intervention and context where implementation will occur [54].
- Access: How the priority population accesses the intervention and any potential barriers to this. Here we have adopted the broad conceptualisation of access as defined by Levesque and colleagues, which includes five dimensions: 1) approachability, 2) acceptability, 3) availability and accommodation, 4) affordability, and 5) appropriateness [72].
- Adaptation: For interventions that are being implemented in a new context, what modifications are needed to improve intervention fit. This should be systematic and documented, and guided by a framework if possible (e.g. the ADAPT guidance or FRAME [54, 73, 74]).

Equity-focused intervention design and adaptation will include meaningful engagement with, and involvement of, community leaders and people with lived experience (i.e. user-centred design), with these processes being community-led where possible. Community engagement has been demonstrated as an important determinant of implementation effectiveness in Indigenous communities [75–77].

##### Identify and involve key stakeholders for implementation

Identify key stakeholders required to ensure equitable implementation, e.g. health professionals, patients and their whānau, community and organisational leaders, community and organisational champions, other stakeholders [51]. Stakeholders should be involved through the subsequent steps.

##### Identify barriers and facilitators to equitable implementation

Identify factors that could influence equitable implementation of the intervention, either positively or negatively [51]. This typically requires engagement with stakeholders and other information gathering processes, e.g. document searches, analysis of local data. An equity-focused determinant framework [48, 78] or another determinant framework with an equity lens applied could be utilised to guide this process. Determinant implementation frameworks identify the factors that enable or inhibit implementation across a range of domains that describe micro-, meso- and macro-level factors [32].

##### Identify measures of implementation and intervention effectiveness

The specific measures that will be monitored and assessed in the subsequent steps to determine effectiveness of the intervention and the implementation pathway. Identifying outcome measures prior to implementation ensures that the pathway design is fit-for-purpose and that the impact of implementing the intervention, based on the selected outcomes, can be evaluated [51, 54].

#### Step 2: Designing the implementation pathway

##### Design communication strategy with equity in mind

Good communication within and between organisations enables knowledge about barriers and facilitators to equitable implementation, strategies and outcomes to be shared [79–84]. The strategy should also include mechanisms for two-way communication between those implementing the intervention and the priority population, and consideration of the ability for patients and their whānau to process, understand and navigate health information and services to engage with the intervention [79].

##### Define resources required for equitable implementation

Resource availability is a key determinant of implementation success [79]. Resourcing for equitable implementation may require different approaches to the setting where evidence of effectiveness was demonstrated. For example, adequate and flexible funding to enable community-based providers to undertake targeted delivery strategies to reach the priority population [79, 83, 85], or adequate numbers of staff who reflect the population served, have the relevant skillset and provide culturally safe care [76, 79, 80, 85].

The type of resources will depend on the intervention and implementation context, but will likely include consideration of staffing (e.g. culturally skilled Māori personnel able to navigate Māori community networks and protocols) and other workforce development, intervention-specific training, cultural safety training, physical resources and financial resources [79].

##### Design equity-specific strategies to address barriers

Design strategies to overcome barriers to equitable implementation identified in the previous step. This may include strategies to overcome accessibility barriers for whānau, implementing cultural safety training for staff, or workforce development strategies to prevent burnout of Māori staff.

##### Design monitoring and evaluation metrics

Establish intervention and implementation pathway-specific measures that will be monitored and evaluated in subsequent steps, including what data will be collected, how it will be analysed and evaluated, and how these results will be shared with stakeholders and utilised while implementation is ongoing. Consideration should be given here to the value of priority population expert input to enable measurement that is robust from priority population perspectives.

#### Step 3: Implementation monitoring

Measuring and monitoring implementation outcomes determined in the previous steps with an equity lens, which may include some or all of the implementation outcomes presented in Table 3. Alternatively, another evaluation framework could be selected (e.g. RE-AIM), with an equity lens applied [86]. For users undertaking Indigenous community-based research, the He Pikinga Waiora Implementation Framework [40] could be used to evaluate outcomes related to community engagement, cultural centredness, integrated knowledge translation and systems thinking; the Framework’s User Guide provides instructions and a guide for conducting evaluation across these dimensions [87].

**Table 3:** Implementation outcomes and health equity considerations. N.B. ‘Priority population’ is used to identify population groups who experience health inequities

**Implementation outcome**	**Definition** (adapted from [88])	**Health equity considerations** (adapted from [51, 54, 86])
Acceptability	Perception of key stakeholders (e.g. health professionals, patients, community members, members of the priority population, other stakeholders) that the intervention is agreeable, palatable or satisfactory	• Is the intervention acceptable for the priority population? • Is the intervention acceptable for improving health equity?
Adoption	Intention, decision or action to implement an intervention	• Is adoption of the intervention equitable across different settings/contexts? If not, why might this be? • Are there any differences between settings relating to adoption of the intervention, e.g. high and low resource settings? • What equity-specific strategies are/could be utilised in the implementation pathway to achieve equitable adoption? And, if tested, how successful were these?
Appropriateness	Perceived fit, relevance, or compatibility of the intervention for a given setting, provider, or consumer, and/or to address a particular issue or problem	• Is the intervention appropriate for the priority population and the implementation setting/context? • Is the intervention appropriate for addressing health inequities? • Is the intervention culturally appropriate?
Penetration/Coverage	Integration of a practice within a service setting	• Is the intervention’s reach, access, service spread or effective coverage (combines coverage and fidelity) equitable for the eligible/priority population(s)? • Who is not reached by the intervention and why? • How can those who are eligible for, but not receiving, the intervention be reached?
Feasibility	Extent to which a new intervention can be successfully used or carried out in a given setting	• Does the intervention allow for health equity barriers to be reduced? • What is the practicability of implementing the intervention in a given setting, especially among priority populations? • What adaptations are required to improve feasibility of intervention implementation for the priority population(s)?
Fidelity	Degree to which an intervention was implemented as originally described or intended	• To what degree is the intervention delivered as intended? • To what degree is implementation equity-focused as originally intended?
Implementation cost	Costs associated with an implementation effort	• What is the cost of implementation in priority populations (and the general population, if applicable)? Are the implementation costs equitable? • What is the final adjusted cost-effectiveness evaluation?
Sustainability	Extent to which a newly implemented intervention is/can be maintained as part of a service’s routine operations	• Is the intervention being equitably maintained/sustained? • In which settings and populations is the intervention being sustained? Do all settings/populations have the capacity and partnerships to sustain intervention delivery? • Are the benefits of the intervention being sustained? Are the sustained benefits equitable? If not, why? • Are health inequities reduced or increased by adaptations to the intervention? • Which strategies (short, medium and long-term) could ensure the maintenance, continuation, durability/embedding or scale up of the intervention in the priority population(s)?

#### Step 4: Outcomes and evaluation

##### Implementation effectiveness

Evaluation of implementation outcomes as per the implementation outcomes measured in the previous step.

##### Intervention effectiveness

Evaluation of effectiveness, as per the measures established in the previous step.

##### Any intervention or implementation pathway adaptations

Evaluation of any specific equity and/or adaptation metrics.

##### Assess overall pathway for inequity causes, solutions and accountabilities

Overall assessment of the intervention’s implementation pathway, including review of any inequities that were identified and the causes, potential solutions and recommendations for adjustments to the pathway and determination of who is responsible and accountable for implementing the changes.

## Discussion

This paper presents an equity-focused implementation framework that is intended to support the design and delivery of equitable implementation pathways for health interventions in Aotearoa NZ. This framework provides step-by-step guidance through the process of undertaking equity-focused implementation research for health programmes, services, and systems [51]. The focus of this adapted framework is on addressing ethnic health inequities, with an initial focus on those experienced by Māori in Aotearoa NZ. It may also have relevance for other Indigenous peoples and other minoritised ethnic or population groups, such as Pacific peoples.

Equitable delivery of health interventions is essential to achieving equitable health outcomes. Conversely, inequitable delivery increases the likelihood that the benefits will not be fully realised for all who are receiving, or intended to receive, the intervention, and may lead to worsening inequities. Equitable implementation ensures the provision of interventions, programmes and services in a fair and just manner that recognises and accounts for systemic social disadvantage and injustice experienced by minoritised population groups [89, 90]. Equitable implementation is said to occur when key equity aspects, including culture, history, values, assets and community needs, are explicitly integrated into implementation science processes, tools and strategies [91]. Process frameworks (often also referred to as models) guide the user through the steps or stages that are needed to translate evidence into routine practice [32]. Few implementation process models are explicitly equity focused [34], although this is changing as the field of implementation science is increasingly attentive to health equity [89, 92–94]. Our framework is intended to complement the He Pikinga Waiora Implementation Framework, the first implementation framework to be developed for the Aotearoa NZ context, by incorporating the key elements of successful and culturally appropriate intervention implementation identified as relevant to Māori and other Indigenous populations (cultural centeredness, community engagement, systems thinking, and integrated knowledge translation) [40].

Adaptation was determined to be appropriate for this study given that, overall, the EquIR conceptual framework provided a clear representation of many aspects that were relevant to meeting the aims of the research. The adapted framework expands on EquIR by incorporating the relevant principles of Te Tiriti o Waitangi as well as refinements that ensure this framework is useable across research and service settings. This included revisions to the foundation of the framework by grounding it in Te Tiriti o Waitangi and Te Ao Māori concepts, and the change of language to be less research-focused. Additionally revising the implementation outcomes to include prompts of equity considerations in relation to the established implementation outcome definitions ensures that the framework can be applied in multiple settings and across diverse population groups.

### Strengths

This study presents an exemplar of the process for adapting an international equity-based implementation framework for the local context in collaboration with an Indigenous (Māori) advisory group and consumer advisory group. The adapted framework is informed by the international implementation science literature on equity and implementation success and incorporates local lessons learned from health equity researchers and stakeholders. The framework is designed to be used in partnership with existing equity tools and approaches, for example, the He Pikinga Waiora Implementation Framework; this offers users the flexibility of incorporating tools and approaches that stakeholders are already familiar with.

### Limitations

As this is a conceptual study, the framework has not been tested and empirical evidence validating its benefits is not yet available. Understanding how the framework performs in practice is an important next step to advancing implementation science in Aotearoa NZ. Additionally, some of the contextual specificity of this framework may limit its use in other populations. In particular, Te Tiriti o Waitangi as the foundation of equitable implementation processes is unique to the Aotearoa NZ context. The evidence informing the framework focuses on addressing ethnic health inequities, particularly those experienced by Māori in Aotearoa NZ. However, this framework could be adapted for use in other population groups who experience health inequities, such as other Indigenous peoples and other minoritised ethnic or population groups. Prospective users in other jurisdictions should be informed by the foundational principles that are relevant to the implementation context or priority group that is intended to benefit from use of the implementation framework.

### Future directions

Operationalisation of this framework in intervention implementation will be important to determine its effectiveness and usefulness in both research and service settings. It is possible that additional refinements may be needed based on the outcomes of the framework’s performance in practice. Furthermore, incorporating an assessment of ‘equity readiness’ for services and/or organisations prior to implementing an intervention is likely to be beneficial. As part of the broader research programme, we have developed an equity readiness assessment tool that can be used alongside the framework for equity-focused implementation work.

We envisage this framework being used by teams planning the implementation of interventions for a variety of ethnic and minority groups, for example the disability sector and LQBT + . Reflection and adaptation may be required to ensure the appropriateness to other groups.

## Conclusion

The framework presented in this study is the first equity-focused process-type implementation framework that has been adapted for the Aotearoa NZ context. The framework is recommended for use by health researchers, service providers and other stakeholders to support the systematic design, delivery, monitoring and evaluation of equitable implementation pathways for health interventions, programmes and services. Future research is needed to determine the effectiveness of utilising this framework to reduce inequities in intervention implementation and achieve equitable outcomes for Māori, Pacific peoples and other minoritised ethnic and population groups.

### Supplementary Information

**Additional file 1: Table 1.** Domains, themes and concepts from qualitative analysis of interviews with researchers and health service leaders about key implementation factors influencing equity.

## Data Availability

Not applicable.

## Abbreviations

CFIR: Consolidated framework for implementation research
EquIR: Equity-based framework for implementation research
FRAME: Framework for reporting adaptations and modifications-expanded
HEAT: Health equity assessment tool
HEIF: Health equity implementation framework
NSC: National science challenge
NZ: New Zealand
RE-AIM: Reach, effectiveness, adoption, implementation and maintenance
TMFs: Theories, models and frameworks

## Glossary

Kaupapa: *Central purpose, initiative, issue*
Hauora: *Holistic health and wellbeing*
Mana Motuhake: *Autonomy, self-determination, sovereignty, self-government*
Tino Rangatiranga: *The fullest expression of rangatiratanga [chieftainship, authority, right to exercise authority, chiefly autonomy, chiefly authority], autonomy, self-determination, sovereignty, self-government*
Whānau: Extended family
Whānau Ora: *A culturally grounded approach to improving the wellbeing of whānau as a group while addressing individual needs*
Te Tiriti o Waitangi: *The Treaty of Waitangi*
