# Supplementary material for: Adapting an equity-focused implementation process framework with a focus on ethnic health inequities in the Aotearoa New Zealand context
Source: Int J Equity Health. 2024 Jan 27;23:15. doi: 10.1186/s12939-023-02087-y (PMC10822165; doi:10.1186/s12939-023-02087-y)
Supplement: Supplementary file 1 — Additional file 1: Table 1. Domains, themes and concepts from qualitative analysis of interviews with researchers and health service leaders about key implementation factors influencing equity. [file 12939_2023_2087_MOESM1_ESM.docx]

Appendix 1

Table 1. Domains, themes and concepts from qualitative analysis of interviews with researchers and health service leaders about key implementation factors influencing equity.

| **Third order domain** | **Second order theme** | **First order concept** | **Definition** |
| --- | --- | --- | --- |
| Macro Environment | Policy | Policy | Refers to the political levers that influence the design and/or implementation process |
| Macro Environment | Funding | Funding arrangements | Relates to any aspect of funding for the design and/or implementation of the initiative |
| Enabling equity environment | Partnership | Community engagement | Engagement with the community in the design of the intervention/research. This is engagement at multiple levels – community leadership, community based organisations, and other stakeholders |
| Enabling equity environment | Partnership | Engaging with te ao Māori | Understanding and incorporating te ao Maori into the ways of working, design and implementation processes |
| Enabling equity environment | Partnership | Whānau engagement and relationships | Connections with the wider whanau as a part of the design and implementation processes |
| Enabling equity environment | Partnership | Whanaungatanga | Getting to know one another and building relationships so effective partnerships and ways of working occur |
| Enabling equity environment | Partnership | Partnership | Organisations and individuals working together to design and implement. |
| Enabling equity environment | Workforce/Training | Recruitment/workforce | The workforce is representative of the local community the intervention is targeted to reach. |
| Enabling equity environment | Workforce/training | Training | The extent of resources and focus on training up the workforce |
| Enabling equity environment | Workforce/training | Task definition | The clarity of roles, responsibilities, and tasks in the delivery of the program |
| Enabling equity environment | Values/attitudes/beliefs | Trust | Refers to the reliance of different roles within the design and implementation processes to be playing their part, and trusting in their relationships |
| Enabling equity environment | Values/attitudes/beliefs | Coordination with other agencies | When services/organisations come together to work in a way that is embedded |
| Enabling equity environment | Values/attitudes/beliefs | Engagement | How different individuals and organisations take interest in one another to facilitate collaboration and support outcomes |
| Enabling equity environment | Values/attitudes/beliefs | Unconscious bias | Reports of cultural bias amongst stakeholders |
| Enabling equity environment | Values/attitudes/beliefs | Beliefs/preferences of the recipient. | Attitudes, beliefs, assumptions of the patient/whānau that impacts on their engagement in the intervention |
| Change readiness | Agreement on the appropriateness | Shared vision | The extent to which there is a common view on the need for the intervention, and the agreement on how appropriate the intervention is |
| Change readiness | Agreement on the appropriateness | Attitudes, assumptions, beliefs | The attitudes, assumptions, beliefs of individual stakeholders about the intervention itself, of the implementation process |
| Change readiness | Championship | Program championship | Activities at all levels to champion the purpose and aims of the program across different stakeholder groups |
| Change readiness | Compatibility | Competing demands | The trade-offs or competing demands that impact on the design and/or implementation process |
| Change readiness | Compatibility | Compatibility | The appropriateness and of the intervention within the organisation’s context |
| Change readiness | Compatibility | Adaptability | The degree to which the intervention can be adapted to the local context, and the mechanisms to support this |
| Change readiness | Compatibility | Clinical demands | Competing demands in the service delivery context meaning ideal processes to address principles of equity are not followed |
| Implementation factors | Leadership | Governance | Relates to how the intervention design and implementation processes are governed. |
| Implementation factors | Leadership | Leadership | Leadership at an organisational level or team level. |
| Implementation factors | Collaborative design | Intervention design | The extent to which the community of interest are involved in the design of the intervention, e.g. part of the design team, in an advisory capacity, in a governance role |
| Implementation factors | Learning and improvement | Feedback and interpretation or process/outcome data | Data examined includes an understanding of the context in which the material is collected from. This includes the ability to interpret the data from the contexts of the lived experience of those targeted. |
| Implementation factors | Learning and improvement | Translation and uptake | Strategies built into the design of the research/intervention to support the translation and uptake of findings in other similar communities. |
| Implementation factors | Learning and improvement | Sustainability | Approaches developed to support the development and scaling of the intervention beyond the initial pilot |
| Implementation factors | Equity outcomes | Equity outcomes (access) | Factors that mention access to health services |
| Implementation factors | Equity outcomes | Equity outcomes (quality) | Data that discusses the experience and the point of care, and the feelings of being safe |
| Implementation factors | Equity outcomes | Equity outcomes (clinical) | Data that talks about the clinical outcomes from the intervention |
| Implementation factors | Team dynamics | Personal relationships | Existing relationships between individuals involved in the design and/or implementation |
| Implementation factors | Team dynamics | Team dynamics | The nature of the individuals making up the team to effect the design and/or implementation processes |
| Implementation factors | Culture | Organisational culture | The embedded culture of an organisation that impacts on the design and/or implementation processes. |
| Implementation factors | Communication | Communication | Communication between different roles and/or organisations to support the design and/or implementation |
| Implementation factors | Communication | Shared decision making | How decision making occurs within the set-up, roll out, and scaling up of the intervention |
| Implementation factors | Adequate resources | Infrastructure | Relates to the physical environment, tools and space, and how it affects the design and/or implementation processes. |
| Implementation factors | Adequate resources | Technical infrastructure | The extent and appropriateness of the technical resources (IT, communication, data capture/sharing) to support the implementation of the intervention. |
| Implementation factors | Adequate resources | Health literacy | The ability of the individual to find, and use information about health services to inform their decision making |
